# Posttranslational Modifications and the Immunogenicity of Biotherapeutics

**DOI:** 10.1155/2016/5358272

**Published:** 2016-04-14

**Authors:** Roy Jefferis

**Affiliations:** Institute of Immunology & Immunotherapy, College of Medical & Dental Sciences, University of Birmingham, Birmingham B15 2TT, UK

## Abstract

Whilst the amino acid sequence of a protein is determined by its gene sequence, the final structure and function are determined by posttranslational modifications (PTMs), including quality control (QC) in the endoplasmic reticulum (ER) and during passage through the Golgi apparatus. These processes are species and cell specific and challenge the biopharmaceutical industry when developing a production platform for the generation of recombinant biologic therapeutics. Proteins and glycoproteins are also subject to chemical modifications (CMs) both* in vivo* and* in vitro*. The individual is naturally tolerant to molecular forms of self-molecules but nonself variants can provoke an immune response with the generation of anti-drug antibodies (ADA); aggregated forms can exhibit enhanced immunogenicity and QC procedures are developed to avoid or remove them. Monoclonal antibody therapeutics (mAbs) are a special case because their purpose is to bind the target, with the formation of immune complexes (ICs), a particular form of aggregate. Such ICs may be removed by phagocytic cells that have antigen presenting capacity. These considerations may frustrate the possibility of ameliorating the immunogenicity of mAbs by rigorous exclusion of aggregates from drug product. Alternate strategies for inducing immunosuppression or tolerance are discussed.

## 1. Introduction

The modern era of biologic therapeutics may be identified with the FDA approval of recombinant insulin (Humulin) in 1982, produced in* E. coli*, and recombinant erythropoietin (EPO) in 1989 (Epogen); since glycosylation of EPO is essential to its function it was, necessarily, produced in a mammalian cell line (a Chinese hamster ovary (CHO) cell line). Despite extensive clinical experience adverse reactions to these recombinant molecules are still encountered, ~2% for insulin [1] and rarer, but more devastating, for EPO [2]. These incidences are frequently due to the patient developing antibodies that are specific for the therapeutic and neutralising its activity (anti-drug antibodies (ADA): anti-therapeutic antibodies (ATA)). The development of ADA suggests that the therapeutic is being recognized as “foreign” (nonself) by the patient's immune system, due to the presence of molecules that exhibit structural features different to those of the endogenous protein/glycoprotein (P/GP). Ironically, a high incidence of ADA is encountered for recombinant antibody therapeutics (mAb/s); this is due, in part, to the fact that each mAb therapeutic is selected for unique epitope specificity and consequently exhibits unique structure.

The starting point for the generation of recombinant P/GPs requires determination of the primary, secondary, and tertiary structure of the endogenous (natural) molecule. This is not a trivial exercise since whilst the gene sequence determines the primary amino acid sequence it does not provide a guide to the precise structure of the active molecule. The nascent polypeptide chain may be subject to cotranslational modifications (CTMs) as it is extruded from the ribosome tunnel, for example, the addition of oligosaccharide; editing for correct folding and initial oligosaccharide processing take place within the endoplasmic reticulum and further posttranslational modifications (PTMs) are effected during passage through the Golgi apparatus. The P/GPs that exit the Golgi may be trafficked within the cell, inserted in the plasma membrane, or secreted into the extracellular environment. The functional activity of a P/GP may be dependent on subsequent chemical modifications (CMs), for example, phosphorylation, and further CMs that constitute its “aging” and subsequent catabolism [3]. It is important to emphasise, therefore, that endogenous (native!) P/GPs exhibit structural heterogeneity. These heterogeneities are compounded when establishing the structure of a purified form of a P/GP because additional heterogeneities are introduced during its isolation, purification, and characterization; in addition the purer the isolated protein is, the lower the yield is and the less certain one can be that it is representative of the active endogenous molecule* in vivo*.

## 2. Overview of Co- and Posttranslational Modifications

Variations in protein structure from that predicted by open reading frame gene sequences may be introduced during transcription and/or translation, by misincorporation at the DNA, RNA, and amino acid level, and the introduction of CTMs [4–10]. Commonly encountered CTMs/PTMs and CMs include glycosylation, phosphorylation, sulphation, glycation, deamidation, and deimination [4, 5]. Additionally, the structural profile,* in vivo*, may vary with age, sex, health, and disease. The human genome contains ~21,000 genes encoding expressed proteins; however, it is estimated that the human proteome may be comprised of 1-2 million protein entities, due to the PTMs/CTMs that are essential to physiological function, systemically, and/or microenvironments [4, 5]. Each human individual should, in theory, be immunologically tolerant to all molecules within their proteome, including all isoforms exhibiting natural PTMs and CMs; however, the exquisite sensitivity of the current generation of assay technologies allows the detection of low affinity antibody to many self-antigens in healthy individuals. Paradoxically, antibodies having the same, or similar, specificity may be amplified in disease states and be a diagnostic marker for individual disease entities [11]. Developments in qualitative and quantitative mass spectrometry during the past decade have “revolutionized” the enumeration of PTMs and CMs generating P/GP heterogeneity and defined >300 structural CTM/PTMs [12–15]. Analysis of 530,264 sequences in the Swiss-Prot database was shown to yield 87,308 experimentally identified PTMs and 234,938 putative PTMs [15]. The potential for structural and functional complexity can be appreciated from the fact that the human genome encodes 518 protein kinases and 200 phosphatases [16, 17]. The second most frequent CTM/PTM is glycosylation. Oligosaccharides may be attached to asparagine residues to generate *N*-linked glycoproteins or to the hydroxyl groups of serine, threonine, or tyrosine to generate* O*-linked glycoforms. The* N*-linked repertoire contains >500 different oligosaccharide structures that may be differentially attached at multiple glycosylation sites to generate >1000 different types of glycan, a consequence of the activities of >250 glycosyltransferases [5, 6, 18, 19].

The potential for complexity/heterogeneity can be illustrated for recombinant mAbs. The full length sequence of an IgG molecule includes ~40 asparagine/glutamine residues; therefore, random deamidation of one of these residues will generate 40 structural variants (isoforms); deamidation of two residues may generate 40 × 40 = 1,560 variants; three: 40 × 1,560 = 59,280 variants; and so forth. When all potential PTMs are considered it has been calculated that a full length IgG molecule may exhibit a heterogeneity embracing 10^8^ isoforms [20]. Recombinant mAbs present a particular challenge since each exhibit a unique structure and must be evaluated on a “case-by-case” basis. Fortunately, current technologies allow for early screening and selection of clones that do not have amino acid residues susceptible to PTM/CTMs within their complimentary determining regions (CDRs); other criteria may be selected to optimise solubility, stability, and so forth. The constant region sequence can also be selected, that is, Ig class and subclass, to define the final drug substance/product developed. A biosimilar candidate must be demonstrated to be structurally and functionally comparable to the innovator product [21, 22].

The starting point for development of a recombinant P/GP therapeutic is the consensus structure of the “wild-type” (WT) molecule. It is required that a candidate recombinant P/GP therapeutic exhibits PTMs comparable to those of the consensus WT structure and an absence of unnatural PTMs introduced by the production process. Structural identity with the WT P/GP may not be possible since production platforms employ nonhuman tissues (CHO, NS0, Sp2/0 cells, etc.) and the secreted P/GP is exposed to the culture medium and products of intact and effete producer cells over an extended period, prior to rigorous downstream purification, formulation, and storage conditions. Any departure from the consensus WT structure may be perceived as nonself by the immune system and result in the generation of ADA. Production in a prokaryotic system (e.g.,* E. coli*) may result in a protein being recovered as an inclusion body that has to be solubilized and refolded* in vitro* to yield a product that may lack natural PTMs or bear unnatural ones. This system may be purposely exploited to develop novel therapeutics, for example, aglycosylated full length antibody molecules that may act as agonists or antagonists but not provoke downstream effector activities. Comparison of the structure of a candidate antibody therapeutic with a WT counterpart is not possible due to the unique structure of its variable regions; however, the amino acid sequence of the constant regions and potential glycoform profiles are established.

Approval of a candidate P/GP therapeutic is dependent on the demonstration of clinical efficacy for a product that has been structurally characterized employing multiple orthogonal physicochemical techniques [23, 24]. The physicochemical characteristics established define the drug substance and drug product and must be maintained throughout the life cycle of an approved drug. Critical Quality Attributes (CQAs) that define drug efficacy are defined [25] and maintained within the production platform developed [26]. These data are the undisclosed intellectual property of an innovator company and it is deemed essentially impossible to produce an identical product employing a similar or alternative platform within another facility; that is, in principle, it is not possible to develop generic biopharmaceuticals. It is possible to introduce improvements in the production process that result in changes in structural parameters if it is demonstrated not to compromise drug efficacy and patient benefit. Importantly, each drug will be assigned a “shelf-life,” that is, a period of time after which physicochemical changes may be evident which impact biologic activity and limit its efficacy and/or prejudice patient benefit. Accelerated storage studies under varying conditions establish structural and functional stability and guide formulation to provide an acceptable shelf-life.

## 3. Immunogenicity

As previously stated endogenous P/GPs may be present* in vivo* in multiple structural isoforms and it may be possible to demonstrate the presence of self-reactive antibodies in serum; however, a healthy individual is functionally tolerant, that is, asymptomatic. Within a disease state the quantitative and qualitative nature of the PTM/CTM repertoire may be amplified with consequent generation of immune complexes and/or aggregated forms that are engulfed by phagocytic cells that have the capacity to process and present antigens, with consequent induction or amplification of an anti-self-response [11, 27–30].

A “casebook” example that may be cited is the anticitrullinated protein response, accepted as the most specific biomarker for rheumatoid arthritis (RA). Citrullinated proteins are generated by the action of peptidylarginine deiminases (PADs), which convert arginine into citrulline in a process called citrullination or deimination [31–33]. This is a natural process; however, in RA, it is amplified and several citrullinated proteins are present within inflamed synovial tissue. It is possible that within the milieu of inflammation some proteins may be denatured and arginine residues that are not normally exposed become accessible to citrullination and may be “seen” as nonself by the immune system. The specificity of this response is reflected in the fact that the diagnostic assay employs a cyclic citrullinated peptide as antigen: the anti-citrullinated peptide antibody (ACPA) response [34, 35]. Importantly, increased levels of ACPA may be detected in advance of clinical manifestations. More recently the presence of anti-carbamylated protein (anti-CarP) autoantibodies have shown specificity for RA and their presence to overlap, at least in part, with ACPA activity: however, anti-CarP-positive and ACPA-negative patients have been described [36–38]. Carbamylated protein arises from the action of cyanate on the epsilon amino groups of lysine residues to generate homocitrulline; cyanate is generated* in vivo* by several routes and its production is enhanced in inflammation.

The original recombinant erythropoietin (EPO) drug (Epogen) was introduced in 1988 and has been used successfully worldwide; however, incidences of neutralising anti-EPO ADA have been reported with the development of pure red cell aplasia (PRCA). With the expiry of the original patent various alternative EPOs have been approved and additional incidences of PRCA reported. A meta-analysis published in 2008 identified 215 cases, worldwide, of ADA and consequent PRCA; 189 of the patients were exposed to Eprex only [39, 40]. In 1998 a “cluster” of PRCA incidences was reported in Europe and investigation revealed variations in formulation of the EPO associated with the absence of human serum albumin (HSA), subcutaneous administration, and capping with an uncoated rubber stopper [40, 41]. It was posited that the net result was likely to be chemical modification and/or aggregation of a critical proportion of the therapeutic; aggregation is considered to be a principle PTM/CM resulting in immunogenicity and the initiation of ADA responses.

Anticipating the patent expiry date of an approved biologic therapeutic the innovator company may develop a variant having enhanced properties and submit for approval and further patent protection. Other biopharmaceutical companies may similarly anticipate patent expiry and seek to develop a copy of the original innovator product, not claimed to be the same but biosimilar, or comparable [25, 26]. Numerous EPO biosimilars have been approved in Europe and the USA and, due to their competitive pricing, have gained market penetration. Copies of EPO have also been generated outside of Europe/USA; however, not all have been rigorously examined by a regulatory authority; others that have not been submitted to a regulatory authority for approval may be marketed as a biosimilar. Cumulative incidences of PRCA have been reported from Thailand where investigations identified EPO products that did not meet international standards and/or were illegal, having been smuggled into the country [41–43].

## 4. Protein Aggregation and Immunogenicity

Early studies investigating the capacity of the immune system to produce specific antibody responses employed potential immunogens of differing molecular weight (MW) and size, from small molecule chemicals to protein antigens and their denatured aggregated forms. These studies suggested a threshold for immunogenicity of ~10 kDa; below this MW immune responses could be elicited if the molecule is presented conjugated to a macromolecule, in which case the chemical is termed a hapten. High MW proteins (e.g., bovine serum albumin; MW ~60 kDa) proved to be immunogenic when delivered together with an adjuvant or in aggregated form. These principles have been validated and extended particularly in relation to vaccine research and development [44].

The biotherapeutics considered in this review are P/GPs having an overall structure (conformation) that confers solubility in aqueous solutions. The integrity (stability) of a P/GP molecule is contributed to by a core of hydrophobic amino acid side chains buried within the internal space of the molecule; however, some such side chains may be only partially buried and form hydrophobic patches on the surface of the molecule. Similarly, most hydrophilic amino acid side chains are exposed on the surface of a molecule but may also be partially buried. Maintenance of structural integrity is essential to function and it is posited that all soluble P/GPs are susceptible to the formation of insoluble fibrillar aggregates under specific denaturing conditions. However, the individual is normally protected by an essential editing function performed within the endoplasmic reticulum (ER) that allows only correctly folded protein to transfer to the Golgi apparatus; misfolded protein activates the unfolded protein response (UPR) and enzymatic degradation within the proteasome [45]. Any disturbance (denaturation) of the native conformation, after secretion, is liable to expose hydrophobic “patches” to aqueous solvent and is compensated for by hydrophobic protein/protein intermolecular interactions, manifest as aggregation [46–48]. The phenomenon of conversion from soluble to insoluble protein may be illustrated by lysozyme, a highly soluble protein that has been studied as a structural model for ~100 years; in spite of its intrinsic solubility it is readily denatured, under nonphysiologic conditions, to form insoluble fibrils (amyloidosis). A more extreme example is the phenomenon of prion disease in which the prion proteins undergo a conformational change,* in vivo*, with the *α* helix portion (54%) of its structure converting to a *β* sheet structure that renders it both insoluble and infectious; that is, it can induce native prion protein to convert to the insoluble/infectious form [48].

From the above it follows that if a P/GP proves to be immunogenic, with the formation of ADA, it is likely due to the presence of nonself forms resulting from denaturation, the presence of inappropriate PTMs/CMs, and/or aggregation. Regulatory authorities have focused particularly on aggregation as being the “bête noire.” Following extensive studies a biologic therapeutic is formulated with excipients selected to minimize denaturation and aggregate formation over the shelf-life of the drug product; it is essential, therefore, to physiochemically characterize the molecular size of drug substance, drug product, and any aggregates present in the approved material and to further monitor these parameters over time [47, 49–55].

## 5. Potential Immunogenicity of Antibody Therapeutics

Clinical experience has shown that even “fully” human mAbs are immunogenic, at least in a proportion of patients [56–64]. An obvious explanation lies with the unique structure of the mAb variable regions that comprise the paratope, formed by the complementarity determining regions (CDRs) that confer unique specificity. The variable regions of recombinant mAb therapeutics are selected from libraries of antibody genes expressed in (i) mice (chimeric); (ii) humanized chimeric mAbs; (iii) phage display libraries generated from an outbred human population; (iv) humanized mice, and so forth. In each case an approved mAb will present unique structural features to an individual recipient (patient) and may be recognized as foreign, with the generation of ADA. Additional nonself structures may be present due to the extensive polymorphism of genes encoding the constant regions of IgG heavy chains and the constant regions of kappa light chains that differs widely in their distribution between ethnic populations [62–65]. To date only one polymorphic form of each therapeutic mAb has been generated and approved; therefore, there will be a high frequency mismatch between the polymorphic variant of a given mAb therapeutic and a proportion of patients. Additionally, since mAbs are manufactured in xenogeneic tissue (hamster, CHO, mouse, NS0, Sp20, etc.), drug substance and drug product may lack the expression of some human CTMs and PTMs whilst expressing others that are not present on human IgGs and may contribute to immunogenicity [11, 56–66].

For the above reasons regulatory authorities demand stringent postapproval pharmacovigilance and reporting of adverse events [67–70]. A considerable literature suggests that the immunogenicity of approved biologics may differ between (i) individual biologics; (ii) manufacturer of the same biologic; (iii) the physical form of the biologic; (iv) patient population; (v) the disease entity; (vi) comorbidities; (vii) doe and route of administration; (vii) period of administration, and so forth. Therefore, each approved mAb therapeutic has to be evaluated on a case-by-case basis. It is of interest to compare clinical experiences from administration of the anti-TNF therapeutics currently approved for the treatment of inflammatory diseases, for example, rheumatoid arthritis, Lupus, and Crohn's disease [59–62]. Whilst the percentage of patients developing ADA may differ between reported studies and disease entities there is general agreement that neutralising antibodies predominate [57, 58, 71]. The presence and specificity of neutralising ADA may be demonstrated by the ability of a patient's serum to inhibit the binding of the mAb to TNF, for example, infliximab; the same patient serum does not inhibit the binding of another anti-TNF mAb, for example, adalimumab mAb, to TNF. Thus if a patient becomes refractive to a given anti-TNF mAb therapeutic, due to the generation of ADA, it may be possible to effect disease remission by switching to an alternative anti-TNF biologic. The formation of ADA may be ameliorated, in a proportion of patients, by coadministration of a mild immunosuppressant, for example, methotrexate [57, 58, 61, 71].

## 6. Aggregation of IgG Antibodies

Monoclonal human IgG antibodies have been available for many decades as myeloma proteins, the products of plasma cell tumours; however, their antigen specificities have not been determined. The plentiful availability of these proteins allowed for antigenic and structural definition of the IgG subclasses, elucidation of Fc receptor recognition specificities, complement activation, and so forth. The first full length sequence of an IgG molecule was determined for an IgG1 myeloma protein [72]. The lack of known antigen specificity did not allow for the generation of antigen/antibody immune complexes (IC); therefore, aggregated forms of IgG were generated as surrogate IC; common protocols included heating at 60–70°C, followed by high speed centrifugation to yield a slightly opalescent solution [73] or cross-linking by bifunctional small molecules 78. Rabbit antisera produced to heat aggregated IgG revealed no new antigenic determinants, compared to normal IgG, but responses to some determinants were heightened [74]. These early findings were corroborated by a recent study of heat aggregated IgGs binding to each of the Fc*γ*R types, expressed as transmembrane molecules on the surface of CHO cells [51].

The demonstration that aggregated forms of IgG could function as surrogate IC has been interpreted in the modern era to suggest that aggregated forms of biologic therapeutics and mAbs in particular may be the principle immunogen triggering the generation of ADA responses. Subsequently, techniques and technologies for qualitative and quantitative characterization of aggregated forms of IgG have been developed and applied to analysis of IgG mAbs subjected to multiple protocols that each mimics conditions experienced by a mAb throughout its production, formulation, storage, and preparation for administration [75–78]. A mouse mAb that was exposed to similar protocols proved to be immunogenic when administered to a naive mouse; the most immunogenic forms were relatively large, insoluble aggregates that exhibited structural features of denatured molecules [79]. It is presumed that aggregates are removed from the circulation by phagocytic cells that degrade them to generate peptides that may be presented by MHC Class II molecules to B and T cells [50].

The propensity for a mAb to aggregate may be determined by the unique structure of the variable regions superimposed on an intrinsic susceptibility of the selected heavy and light chain isotypes. An analysis of the constant regions of an IgG1/kappa molecule identified partially exposed hydrophobic side chains that when in proximity to other hydrophobic residues constitute aggregation prone regions (APRs) (“spatial-aggregation-propensity (SAP)”) [80]. APRs were identified within the CH1, hinge, CH2, and CH3 domains of the IgG1 heavy chain and the constant regions of both kappa and lambda light chains; substitution of targeted hydrophobic amino acids with selected hydrophilic residues generated more stable proteins with a diminished tendency to aggregate. The APRs identified in IgG1 molecules are present also in the IgG2, IgG3, and IgG4 subclass proteins [80–82]. The presence of “open” or “free” cysteine residues is another parameter shown to result in the generation of aggregates, through intermolecular disulphide bond formation, under differing stress conditions, and is particularly relevant for IgG2 subclass proteins [83]. As expected the individual variable sequences can have a profound impact on susceptibilities to aggregation. The germline sequences for VH, VK, and VL have been comprehensively reviewed [84] and analysed for the aggregation potential of their protein products [85]. These criteria may now be applied to the selection of clones producing potential IgG therapeutics. Close interrogation of the immunogenicity of currently approved mAbs and biologics may reveal further parameters that contribute to immunogenicity.

Recent studies have shown that the structure/form of a mAb therapeutic must be considered beyond that of the formulated drug product received by the pharmacy. Instructions to pharmacists for resuspension of the mAb therapeutics Herceptin and Avastin, for intravenous administration, specified the use of 0.9% saline and specifically excluded of the use of 5% dextrose solutions; no reason was given [86, 87]. The consequences for contravening this instruction demonstrated that titration of either Herceptin or Avastin in 5% dextrose into human plasma or serum resulted in the formation of insoluble aggregates [87]; possibly resulting in adverse reactions on administration to patients and/or sensitising them to later production of ADA. The phenomenon was further investigated and it was shown that addition of a 5% dextrose solution, at pH 6.0–6.2, to plasma/serum resulted in the formation of aggregates of complement components that then bound the mAb [88]. It was noted that instructions for resuspension of Remicade specified 0.9% saline, pH 7.2, conditions that did not result in the formation of complexes; similarly, neither did Herceptin or Avastin; however, these mAbs exhibit other instabilities at higher pH values [87–89]; for example, Asn 30 of Herceptin deamidates at pH > 5.0, which lowers product bioactivity [4, 88].

## 7. Formation and Removal of Immune Complexes (ICs)

It is axiomatic that an IgG antibody binds its target antigen (pathogen!) to form an antigen/antibody immune complex (IC). Similarly, it is axiomatic that the IC has to be removed and destroyed. This is accomplished by cells that bear receptors specific for the Fc region (Fc*γ*R) of the IgG molecule (IgG-Fc) resulting in uptake and consequent destruction within lysosomes. The peptides generated may bind to MHC Class II molecules and subsequently be expressed on the cell surface for presentation to helper CD4 T cells. This promotes and amplifies a humoral immune response [90]. The integrity of the individual is dependent on tolerance to self-molecules; however, tolerance has been shown to be a dynamic ongoing process with activation/anergy being dependent on the strength of binding of peptide bearing MHC II molecules and helper T cells. Consequently, self-reactive T cells and antibodies, usually of IgM isotype, can be enumerated in normal healthy individuals.

The above summarizes the body's protective response to “foreign bodies” (pathogens) that gain access to tissue or vascular sites. The aim of delivering a mAb therapeutic to a patient is for it to bind its target antigen (self-molecule) with the formation of IC that will activate mechanism of removal and destruction similar to those activated by aggregated P/GPs. I pose the following question therefore.

*What Is the Difference between Aggregates and Immune Complexes*? An early study employed a matches set of recombinant mAbs to evaluate the ability IC of each human antibody class and subclass to trigger the neutrophil respiratory burst; presumed to act through IgG-Fc receptors (Fc*γ*RI, Fc*γ*RIIA, and Fc*γ*RIIIB), in both the presence and absence of complement [91, 92]. This and other studies demonstrated different outcomes for each antibody isotype depending on the epitope density and the antibody/antigen ratio at which ICs were formed [28, 29, 93]. A further refinement has monitored the binding of ICs, formed with antigens having differing epitope densities, with a panel of CHO cells each expressing a single Fc*γ*R type. This study also demonstrated that the avidity of binding to Fc*γ*R increased with epitope density and revealed a different order of Fc*γ*R binding from that reported for studies of monomeric mAb binding to Fc*γ*R, [51]; that is, the “received” Fc*γ*R binding specificities of the IgG subclasses widely reported that the IgG subclasses are not an accurate guide to the specificities of their IC* in vivo*. The fine epitope specificity of a mAb can also have a determining impact on the structure of the IC formed and resulting MoA, as illustrated by approved anti-CD20 mAbs. Anti-CD20 mAbs are classified as Type I or Type II depending on whether or not they trigger redistribution of cell surface CD20 into lipid rafts; Type I do so and as a result are able to activate the complement cascade whilst Type II bind to individual CD20 tetramers only and do not activate complement [94, 95]. The extracellular domain of the CD20 molecule is comprised of only ~40 amino acids residues and crystallography demonstrates that Type I and Type II antibodies bind overlapping nonidentical epitopes.

A further example is provided by anti-TNF mAbs. Soluble TNF exists as a trimer and is potentially trivalent for mAb binding and able to form three-dimensional immune complexes with divalent antibody. A study of the size distribution of immune complex formed between TNF and the approved anti-TNF biologics infliximab and etanercept, at differing antigen/antibody ratios, showed that each antibody generated immune complexes having a unique size profile [96]. It may be presumed therefore that they will differ in their Fc*γ*R activating properties. It has been suggested that a fundamental difference exits between IgG-ICs and aggregated IgG in that the CDRs of the former are engaged but they are exposed in the latter; however, X-ray crystal structural analysis of Fab-antigen complexes shows that, for the majority of complexes analysed, not all CDRs are engaged in antigen binding [97]. The above parameters may be compounded by the fact that, in contrast to most recombinant biologics, mAbs are delivered at high doses (~400 mg); therefore, an unnatural or degraded nonself component present at a level of 0.001% can constitute a viable immunogenic dose [9, 98].

## 8. Chemical Modifications (CMs) of Amino Acid Side Chains

*N- and C-Terminal Residues*. Unique N-terminal sequence may be obtained for the heavy and light chains of most monoclonal IgG paraprotein; however, for some, the N-terminal amino acid yield may not be quantitative or may appear to be entirely “blocked” [99]. This results when a gene encodes for the incorporation of N-terminal glutamic acid or glutamine residues that may subsequently cyclize,* in vivo* and/or* in vitro*, with the generation of pyroglutamic acid (pGlu) [99–104]. The formation of pGlu in antibodies [101] and therapeutic proteins is a concern for the biopharmaceutical industry since it introduces charge heterogeneity and variations may be considered to be evidence for lack of process control [18]. Importantly, N-terminal pGlu is also implicated in Alzheimer's disease and dementia since it increases the tendency for proteins to form insoluble fibrils; light chains are particularly prone to the formation of pGlu fibrils [101, 105, 106]. As there is no evidence of benefit attached to the presence of N-terminal pGlu, to either the heavy or light chain, it may be best to select against its presence, where possible, during clone selection for a potential mAb therapeutic.

Sequencing studies reported the C-terminal residue of serum derived IgG heavy chains to be glycine; however, the IgG subclass genes encode a C-terminal lysine residue. It was later shown that the lysine residue is cleaved,* in vivo*, by an endogenous carboxypeptidase B. Recombinant IgG molecules produced in mammalian cells exhibit mixed populations of molecules with lysine present or absent on each heavy chain and subsequent charge heterogeneity [107]. A concept paper produced by the EBE (European Biopharmaceutical Enterprises), a specialized group of EFPIA (European Federation of Pharmaceutical Industries and Associations), included the statements: “A number of scientific publications suggest that C-terminal lysine truncation has no impact on biological activity, PK/PD, immunogenicity and safety.” And elsewhere in the document: “Lysine truncation does not appear to adversely affect product potency or safety. However, taking a conservative approach potential C-terminal lysine effects on all antibodies cannot be ruled out. Thus, lysine truncation should be characterized, and process consistency should be demonstrated during product development; regulatory agencies suggest that C-terminal lysine content should be reported for both the characterization and development phases” [108]. Removal of C-terminal lysine results in the presence of a C-terminal glycine residue that, when produced in CHO cells, may be subject to amidation, introducing further structural and charge heterogeneity [109]. A recent report demonstrated that this has been circumnavigated by genetically engineering CHO cells to “knock-down” expression of the peptidylglycine *α*-amidating monooxygenase (PAM) enzyme [110]. The above comments and recommendations are contradicted by a recent study that claimed that IgG with C-terminal lysine constitutes a preform of the molecule that prevents the formation of IgG hexamers that can activate the complement cascade [111].

## 9. Cysteine and Disulphide Bond Formation

The gene sequence for the human IgG1 subclass protein Eu encodes for 5 light chain and 9 heavy chain cysteine residues, that is, 28 for the H2L2 heterodimer. The standard structural cartoon for the human IgG1 protein (Eu) exhibits 12 intrachain and four interchain disulphide bridges. This general pattern of intrachain disulphide bridge formation is maintained for each of the IgG subclasses; however, the number of interchain bridges and their architecture vary between and within the IgG subclasses [72, 112–118]. Heterogeneity of disulphide bridge formation has been reported for normal serum derived IgG, myeloma proteins, and recombinant mAbs. Formation of the H2L2 dimer occurs following release of heavy and light chains into the endoplasmic reticulum (ER), with evidence that binding of the constant region of the light chain (C_L_) to the heavy chain C_H_1 domain “catalyzes” the generation of a correctly folded H2L2 structure [112]. This nascent form explores multiple dynamic structures, with the formation of native and nonnative disulphide bonds that are transiently formed and reduced until a low energy conformation is achieved [112, 113]; it should be noted that little or no processing of the high mannose oligosaccharide will have occurred at this point; therefore, the conformation of the secreted IgG-Fc will not be achieved until oligosaccharide processing is completed.

The IgG1 molecule establishes the “standard” pattern with two inter-heavy chain disulphide bridges and a single light-heavy chain bridge; IgG2, IgG3, and IgG4 express 3, 11, and 2 inter-heavy chain bridges, respectively. The cysteine residues that form interchain disulphide bridges are clustered within the hinge region and may be subject to reduction and reformation when present in a reducing environment. Heterogeneity in disulphide bond formation in IgG2 was first reported for recombinant IgG2 proteins but, later, observed for normal serum derived IgG2 also [114–118]. The interconversion of these isoforms is dynamic and promoted by a reducing environment provided by the presence of thioredoxin reductase, released into culture media by effete cells; it can be ameliorated by control of dissolved oxygen levels [114–118]. An* in vitro* model revealed that susceptibility to reduction/oxidation differed between IgG subclasses and light chain types with sensitivity being in the order IgG1*λ* > IgG1*κ* > IgG2*λ* > IgG2*κ* [116].

A core hinge region sequence of -Cys-Pro-Pro-Cys-, present in IgG1, IgG2, and IgG3, forms a partial helical structure that does not allow for intra-heavy chain disulphide bridge formation; however, the homologous sequence in the IgG4 subclass is -Cys-Pro-Ser-Cys- and this does allow for intra-heavy chain disulphide bridge formation. Consequently, natural and recombinant IgG4 antibody populations are a mixture of molecules exhibiting inter- and intrahinge heavy chain disulphide bridge isoforms [118–122]. The IgG4 form having intrahinge heavy chain bridges is susceptible to dissociation into half-molecules (HL) that may reassociate randomly to generate bispecific molecules; that is, a molecule that is monovalent for two nonidentical antigens (epitopes); this phenomenon is referred to as “Fab arm exchange.” The exchange is also facilitated by presence of an arginine residue at position 409 (R409) in the IgG4 heavy chain that reduces noncovalent CH3/CH3 interactions, relative to the presence of lysine 409 (K409) present in IgG1, IgG2, and IgG3 molecules. Lateral noncovalent interactions between the two C_H_3 domains of R409 IgG4 are reduced such that, under physiologic conditions and in the absence of hinge region intra-heavy chain disulphide bridges, they dissociate to form HL heteromonomers; a polymorphic variant of IgG4 exists that has K409 residue and is not subject to Fab arm exchange [120–122].

## 10. Oxidation of Methionine

Methionine residues exposed, or partially exposed, on the surface of native or denatured proteins may be susceptible to oxidation. Methionine residues within variable region framework sequences of mAbs have not been reported to be vulnerable to oxidation but residues exposed within CDRs have been [123–127]. Consequently, sequencing of prospective clones is advised to inform selection and rejection of clones having methionine within CDRs. It has been shown that methionine residues M252 and M428 of IgG1 and IgG2 subclass proteins are susceptible to oxidation [123]. Although these residues are distant from each other in linear sequence, they are conformationally proximal, at the C_H_2/C_H_3 interface. The interaction site for the neonatal Fc receptor (FcRn), SpA, and SpG is similarly localised to the C_H_2/C_H_3 interface and M252 and M428 oxidation has been shown to reduce the affinity of binding to these ligands and to reduce catabolic half-life [124–127]. Minimal levels of M252 oxidation (2–5%) are reported for IgG mAbs held in formulation buffers, whilst lower levels of oxidation are reported for M428; however, oxidation of these residues increases under conditions of accelerated stability testing and on prolonged storage [123]. Analysis of Herceptin, obtained from a pharmacy, and a potential biosimilar demonstrated that care has to be exercised when resuspending this mAb therapeutic since a discrepancy was observed for the level of M252 oxidation between the innovator product (4.39%) and the proposed biosimilar (10.33%) [128].

## 11. Deamidation: Asparagine and Glutamine

Deamidation of asparagine and glutamine residues generates aspartic acid, isoaspartic acid, or glutamic acid, respectively, and is a frequently encountered PTM [9, 10, 129, 130]. Deamidation of asparagine residues is influenced by adjacent amino acid residues, particularly the presence of a glycine residue C-terminal to the asparagine [-N-G-] and the degree of exposure to external environments. Studies of IgG1 and IgG2 proteins,* in vitro* and* in vivo*, have shown that asparagine residues 315 and 384 are susceptible to deamidation with the formation of isoaspartic and aspartic acid residues, respectively [129–134]. The relative susceptibility to deamidation at these sites varied between studies; however, the significance may be ameliorated by the finding that ~23% of asparagine 384 residues of normal polyclonal IgG are deaminated to aspartic acid; thus it may be assumed that healthy humans are constantly exposed to IgG bearing this PTM and that it might be considered to be a “self” structure. These studies did not identify asparagine deamidation within the constant region of the kappa light chains.

By contrast deamidation within variable regions, particularly within CDRs, of recombinant antibodies has been shown to compromise antibody specificity and/or binding affinity [131–134]. Interestingly, the approved blockbuster antibody therapeutic Trastuzumab (Herceptin) has asparagine residues in light chain CDR1 (Asn 30) and heavy chain CDR2 (Asn 55) that were shown to be susceptible to deamidation on accelerated degradation studies [4, 13]; the approved drug substance did not exhibit deamidation of these residues; therefore, their presence or absence could be used as a lot release criterion [134]. As previously discussed the levels of deamidation of asparagine residues, both within variable and constant regions, of a proposed Herceptin biosimilar were higher than that reported for the innovator molecule [128] underlining the susceptibility to deamidation of these residues and the care that has to be exercised when resuspending this antibody therapeutic.

Glutamine residues are relatively resistant to deamidation and no glutamine residues were reported to be subject to deamidation under nondenaturing conditions [8, 13]. Under conditions of accelerated degradation six Gln residues of a mAb were shown to be susceptible to deamination: four in variable regions and residues 295 and 418 in the IgG-Fc [8]. The cyclization of N-terminal glutamine to form pyroglutamic acids has been discussed, above.

## 12. *γ*-Carboxylation and *β*-Hydroxylation

The function of proteins of the blood coagulation system may be dependent on *γ*-carboxylation and *β*-hydroxylation [135–138]. Both PTMs contribute to the binding of calcium ions and are important, and in some cases essential, for blood factors VII, IX, and X, activated protein C, and protein S of the anticoagulant system. These proteins are comprised of structurally distinct domains with the N-terminal Gla (gamma-carboxyglutamic acid-rich) domain providing *γ*-carboxylation sites and the EGF (epidermal growth factor-like) domains the *β*-hydroxylation sites. Typically Gla domains are approximately 45 amino acids long and contain 9–12 Gla residues. Carboxylation of Gla domain glutamate residues is not dependent upon occurrence within a specific consensus sequence, but carboxylase binding is mediated by an immediately adjacent propeptide region, which is subsequently removed by proteolysis [135–137].

Hydroxylation of target EGF domain aspartate or asparagine residues is catalyzed by a *β*-hydroxylase located in the ER. EGF domains are ~45 amino acids long and contain one potential hydroxylation site. Hydroxylation is consensus sequence dependent and is usually partial, with only a fraction of target molecules being hydroxylated. Full carboxylation and hydroxylation, on the other hand, are essential to maintaining biological activity of protein C [136–139]. The native molecule displays nine carboxylation and one hydroxylation sites. Such stringent PTM requirements could not be met by CHO cells, forcing the developers of the recombinant proteins of the coagulation system to develop a modified human cell line (HEK 293) for their manufacture [138].

## 13. Sulphation

Sulphation is a PTM predominantly associated with secretory and membrane proteins [140]. The attachment of a sulphate (SO^3−^) group to an oxygen atom of tyrosine, serine, or threonine residues is effected by a sulfotransferases enzyme present in the* trans*-Golgi network [141]. In the context of biopharmaceuticals native hirudin (a leech-derived anticoagulant) and blood factors VIII and IX are usually sulphated. Neither of the approved recombinant forms of hirudin are sulphated although it has been shown that sulphated hirudin (at Tyr63) displays 10-fold tighter affinity for thrombin than do unsulphated analogues [142]. Whilst over 90% of native factor IX molecules are sulphated, less than 15% of the approved recombinant form are, with apparently little if any difference in product efficacy [143]. Sulphation of factor VIII is required for optimal binding to its plasma carrier protein (von Willebrand's factor) and, interestingly, people inheriting a factor VIII Tyr1680 → Phe mutation often display mild haemophilia [140]. Several hormone cell surface receptors are known to be tyrosine sulphated, and sulphation is required for high affinity ligand binding and subsequent receptor activation [144, 145].

## 14. Glycosylation: Immunogenic Glycoforms Produced by Rodent Cell Lines

The impact of glycosylation on secretion, stability, function, and immunogenicity of recombinant GPs remains a focal point within the biopharmaceutical industry. The glycoform profile of a GP may be species, cell type, and, possibly, sex specific. Recombinant EPO (rEPO) when first produced in CHO cells exhibited increased biologic activity compared to the natural product* in vitro*; however,* in vivo* studies showed a very low level of biological function. It was shown that this was due to rapid clearance in the liver, via the asialo glycoprotein receptor, due to the oligosaccharide chains not being “capped” with terminal sialic acid residues. Successful generation of appropriately sialylated rEPO was achieved and Epogen was approved in 1989 [2]. Since that time many improvements have been introduced, including protein engineering to introduce additional glycosylation sites [146, 147]. The nonhuman production platforms employed, for example, CHO, NS0, and Sp2/0 cells which may add sugar residues that are foreign to humans and consequently confer immunogenicity.

The prototype IgG antibody molecule bears oligosaccharide N-linked to asparagine residue 297 of the IgG-Fc heavy chain. Although the presence or absence of IgG-Fc oligosaccharide does not affect antigen binding specificity, it has been reported to modulate binding affinity. Its main impact is to modulate activation of downstream effector functions that eliminate and destroy antibody/pathogen ICs; mAb therapeutics may be “customised” to activate the same effector mechanisms for the elimination of cancer cells, and so forth [11, 148]. The oligosaccharide released from normal polyclonal IgG-Fc is heterogeneous and essentially comprised of the core heptasaccharide with the variable addition of fucose, galactose, bisecting N-acetylglucosamine, and N-acetylneuraminic (sialic) acid residues [9, 11, 148–150]. Early analytical studies revealed variations in the content of galactose residues and G0 (zero galactose), G1, and G2 glycoforms were enumerated; however, it was later shown that whilst a majority of IgG-Fc oligosaccharides bore fucose residues, a significant proportion did not; therefore a revision of glycoform designations was required; thus G0, G1, and G2 refer to IgG-Fc oligosaccharides that do not include fucose, whilst G0F, G1F, and G2F refer to oligosaccharides bearing both fucose and galactose; when bisecting N-acetylglucosamine is present a B is added, for example, G0B, G0BF, and G1BF; sialylation at the galactose residues is designated as G1FS, G2FBS, and so forth. The approximate composition of neutral oligosaccharides released from normal polyclonal human IgG-Fc is G0: 3%; G1: 3%; G2: 6%; G0F: 23%; G1F: 30%; G2F: 24%; G0BF: 3%; G1BF: 4%; and G2BF: 7% [9, 11, 22–24, 148–151]. It is important to define the glycoform of the intact IgG molecule, for example, [G0/G1F] and [G1F/G2BF], since it has been shown that individual IgG molecules may be comprised of symmetrical or asymmetrical heavy chain glycoform pairs [152, 153]. Recombinant mAbs expressing each of the naturally occurring IgG-Fc glycoforms have been generated to determine their respective abilities to activate a range of effector mechanisms; in addition truncated, aglycosylated, and novel glycoforms have been generated that have contributed to our understanding of the role of glycosylation in the interactions of IgG-Fc with effector ligands [11, 22, 148–155].

The glycosylation profile of each approved mAb therapeutic is identified as a CQA, whether the aim is to optimise or minimize effector function potential, that is, the MoA. The first criterion is therefore to produce a mAb having either 100% or 0% oligosaccharide occupancy. The CHO, NS0, and Sp2/0 cell lines used for the production of mAbs produce predominantly G0F heavy chain glycoforms with relatively low levels of galactosylated and nonfucosylated IgG-Fc, relative to normal polyclonal IgG-Fc; however, precise culture conditions may impact the glycoform profile of the product [22, 156–159]. Unfortunately, these production cell lines may also add sugars that are not expressed on human glycoproteins and may be immunogenic. Thus, whilst CHO cell lines may add N-acetylneuraminic acid residues they do so in *α*(2-3) linkage, rather than the *α*(2–6) linkage present in human IgG-Fc. A particular concern is that the addition, by NS0 and Sp2/0 cells, of galactose in *α*(1–3) linkage to galactose linked *β*(1–4) to the N-acetylglucosamine residues [160–163]. Humans and higher primates do not have a functional gene encoding the transferase that adds galactose in *α*(1–3) linkage; however, due to continual environmental exposure to the gal *α*(1–3) gal epitope, for example, in red meats, humans develop IgG antibodies specific to this antigen; a proportion of individuals develop IgE antibodies and incidences of immediate hypersensitivity reactions have been reported, some resulting in death [160, 161]. The gal *α*(1–3) gal epitope is widely expressed on hamster cells and some derived CHO cell lines have been shown to be capable of (gal *α*(1–3) gal) addition [163]. Similarly, CH0, NS0, and Sp2/0 cells may add an N-glycolylneuraminic acid, in *α*(2-3) linkage that also may be immunogenic in humans [156–163]. A significant population of normal human IgG-Fc bears a bisecting N-acetylglucosamine residue that is absent from IgG-Fc produced in CHO, NS0, or Sp2/0 cells. Studies of homogeneous IgG-Fc glycoforms, generated* in vitro*, have shown qualitative and quantitative differences in effector function activities between the IgG subclasses and for differing glycoforms within each subclass [11, 64, 164–166]. It has not proved possible to manipulate culture medium conditions to generate predetermined homogeneous mAb glycoform profiles; however, significant “tweaking” of the profile can be achieved during a production run [158, 159] and cellular engineering has been employed to enhance production of particular human IgG-Fc glycoforms.

## 15. Summary and Conclusions

The thrust of this review is that natural P/GPs are structurally heterogeneous and comprised of multiple isoforms, due to variations in CTMs, PTMs, and CMs. The isoform composition of the proteome may differ between individuals and, over time, within individuals; however, the integrity of the individual is maintained by functional immunological tolerance. The production of a recombinant P/GP in a heterologous system will inevitably result in the generation of isoforms structurally different to endogenous P/GPs that may be perceived by the immune system as nonself and result in an immune response with the production of ADA that compromise therapeutic benefit and/or induce adverse events. Recombinant mAbs presents a particular challenge because an endogenous molecule is not available for structural comparison; that is, they are, by definition, structurally and functionally unique. Multiple parameters that may impact on immunogenicity have been discussed with a particular emphasis on denaturation leading to the formation of immunogenic aggregates. Whilst this has validity for recombinant P/GP therapeutics in general, it may not be so evident for mAbs since their MoA depends on the formation of ICs that are themselves aggregates. The size and architecture of IC aggregates (ICA) formed are dependent on multiple parameters, including affinity and antigen/mAb ratio.

It is held that on chronic exposure all recombinant P/GPs are immunogenic, at least in a proportion of patients; however, the incidence and consequences of immunogenicity may vary depending on the disease treated. Treatments for cancer include drugs that target dividing cells with the inevitable collateral consequence of compromising the immune system, that is, immunosuppression; thus short term exposure to a mAb in the treatment of cancers may not occasion the generation of ADA. In contrast treatment of chronic diseases with mAb may result in repeated episodes of remission and relapse over extended time periods and has been shown to increase the incidence of development of ADA. A palliative measure may be to induce a low level of immunosuppression. This has been realized for rheumatic diseases with exposure to the mild immunosuppressant methotrexate and anti-TNF mAb [167, 168]. Early studies established that immunological tolerance can be induced experimentally by exposure to aggregate free forms of a potential immunogen [169, 170]; this potential has been exploited clinically to reduce the incidence of ADA formation in patients receiving the mAb Alemtuzumab. A single amino acid mutant of the antibody was generated that resulted in loss of antigen binding activity 178. Exposure of patients to a high dose of this mutant prior to dosing with the active antibody reduced the incidence of immunogenicity from ~74% to 21% [29, 170, 171]. The crucial difference between Alemtuzumab and the mutant was that the latter is not able to form ICs, thus demonstrating the generation of ICs as a parameter contributing to immunogenicity; that is, the outcome of exposure to ICs and aggregates may be equivalent. This suggests that in addition to characterizing aggregated forms of mAb in drug product, assumed to be present at the time of administration, studies of the structure and function of ICs formed on administration of mAb should be investigated [86–88]; the necessary tools are now available [172–176].

## References

[1] Ghazavi M. K., Johnston G. A. (2011). Insulin allergy. *Clinics in Dermatology*.

[2] MacDougall I. C., Roger S. D., De Francisco A. (2012). Antibody-mediated pure red cell aplasia in chronic kidney disease patients receiving erythropoiesis-stimulating agents: new insights. *Kidney International*.

[3] Welle S. (1999). *Human Protein Metabolism*.

[4] Harris R. J., Kabakoff B., Macchi F. D. (2001). Identification of multiple sources of charge heterogeneity in a recombinant antibody. *Journal of Chromatography B: Biomedical Sciences and Applications*.

[5] Zhong X., Wright J. F. (2013). Biological insights into therapeutic protein modifications throughout trafficking and their biopharmaceutical applications. *International Journal of Cell Biology*.

[6] http://proteomics.cancer.gov/whatisproteomics.

[7] Jefferis R. (2012). Isotype and glycoform selection for antibody therapeutics. *Archives of Biochemistry and Biophysics*.

[8] Goetze A. M., Liu Y. D., Arroll T., Chu L., Flynn G. C. (2012). Rates and impact of human antibody glycation in vivo. *Glycobiology*.

[9] Khawli L. A., Goswami S., Hutchinson R. (2010). Charge variants in IgG1: isolation, characterization, in vitro binding properties and pharmacokinetics in rats. *mAbs*.

[10] Wang W., Singh S., Zeng D. L., King K., Nema S. (2007). Antibody structure, instability, and formulation. *Journal of Pharmaceutical Sciences*.

[11] Burska A. N., Hunt L., Boissinot M. (2014). Autoantibodies to posttranslational modifications in rheumatoid arthritis. *Mediators of Inflammation*.

[12] Chicooree N., Unwin R. D., Griffiths J. R. (2015). The application of targeted mass spectrometry-based strategies to the detection and localization of post-translational modifications. *Mass Spectrometry Reviews*.

[13] Lanucara F., Eyers C. E. (2013). Top-down mass spectrometry for the analysis of combinatorial post-translational modifications. *Mass Spectrometry Reviews*.

[14] Farriol-Mathis N., Garavelli J. S., Boeckmann B. (2004). Annotation of post-translational modifications in the Swiss-Prot knowledge base. *Proteomics*.

[15] Khoury G. A., Baliban R. C., Floudas C. A. (2014). Proteome-wide post-translational modification statistics: frequency analysis and curation of the swiss-prot database. *Scientific Reports*.

[16] Manning G., Whyte D. B., Martinez R., Hunter T., Sudarsanam S. (2002). The protein kinase complement of the human genome. *Science*.

[17] Sacco F., Perfetto L., Castagnoli L., Cesareni G. (2012). The human phosphatase interactome: an intricate family portrait. *FEBS Letters*.

[18] Stanley P. (2011). Golgi glycosylation. *Cold Spring Harbor Perspectives in Biology*.

[19] Marth J. D., Grewal P. K. (2008). Mammalian glycosylation in immunity. *Nature Reviews Immunology*.

[20] Kozlowski S., Swann P. (2006). Current and future issues in the manufacturing and development of monoclonal antibodies. *Advanced Drug Delivery Reviews*.

[21] http://www.fda.gov/Drugs/DevelopmentApprovalProcess/%20HowDrugsareDevelopedandApproved/ApprovalApplications/TherapeuticBiologicApplications/ucm113522.htm.

[22] EMA http://www.ema.europa.eu/docs/en_GB/document_library/Scientific_guideline/2009/09/WC500003074.pdf.

[23] http://www.fda.gov/downloads/Drugs/.../Guidances/ucm073507.pdf.

[24] http://www.ema.europa.eu/ema/index.jsp?curl=pages/regulation/document_listing/document_listing_000162.jsp.

[25] FDA (2014). *Guidance for Industry: Scientific Considerations in Demonstrating Biosimilarity to a Reference Product*.

[26] http://www.ema.europa.eu/ema/index.jsp?curl=pages/special_topics/document_listing/document_listing_000318.jsp.

[27] Gatti E., Pierre P. (2003). Understanding the cell biology of antigen presentation: the dendritic cell contribution. *Current Opinion in Cell Biology*.

[28] Walsh G., Jefferis R. (2006). Post-translational modifications in the context of therapeutic proteins. *Nature Biotechnology*.

[29] Jefferis R. (2011). Aggregation, immune complexes and immunogenicity. *mAbs*.

[30] Mastrangelo A., Colasanti T., Barbati C. (2015). The role of posttranslational protein modifications in rheumatological diseases: focus on rheumatoid arthritis. *Journal of Immunology Research*.

[31] Pruijn G. J. M. (2015). Citrullination and carbamylation in the pathophysiology of rheumatoid arthritis. *Frontiers in Immunology*.

[32] Shelef M. A., Sokolove J., Lahey L. J. (2014). Peptidylarginine deiminase 4 contributes to tumor necrosis factor *α*-induced inflammatory arthritis. *Arthritis and Rheumatology*.

[33] Zhao X., Okeke N. L., Sharpe O. (2008). Circulating immune complexes contain citrullinated fibrinogen in rheumatoid arthritis. *Arthritis Research and Therapy*.

[34] van der Linden M. P. M., van der Woude D., Ioan-Facsinay A. (2009). Value of anti-modified citrullinated vimentin and third-generation anti-cyclic citrullinated peptide compared with second-generation anti-cyclic citrullinated peptide and rheumatoid factor in predicting disease outcome in undifferentiated arthritis and rheumatoid arthritis. *Arthritis and Rheumatism*.

[35] Jilani A. A., Mackworth-Young C. G. (2015). The role of citrullinated protein antibodies in predicting erosive disease in rheumatoid arthritis: a systematic literature review and meta-analysis. *International Journal of Rheumatology*.

[36] Brink M., Verheul M. K., Rönnelid J. (2015). Anti-carbamylated protein antibodies in the pre-symptomatic phase of rheumatoid arthritis, their relationship with multiple anti-citrulline peptide antibodies and association with radiological damage. *Arthritis Research and Therapy*.

[37] Chimenti M. S., Triggianese P., Nuccetelli M. (2015). Auto-reactions, autoimmunity and psoriatic arthritis. *Autoimmunity Reviews*.

[38] Willemze A., Toes R. E. M., Huizinga T. W. J., Trouw L. A. (2012). New biomarkers in rheumatoid arthritis. *Netherlands Journal of Medicine*.

[39] Macdougall I. C., Casadevall N., Locatelli F. (2015). Incidence of erythropoietin antibody-mediated pure red cell aplasia: the Prospective Immunogenicity Surveillance Registry (PRIMS). *Nephrology Dialysis Transplantation*.

[40] Locatelli F., Del Vecchio L., Pozzoni P. (2007). Pure red-cell aplasia ‘epidemic’—mystery completely revealed?. *Peritoneal Dialysis International*.

[41] Wish J. B. (2011). Erythropoiesis-stimulating agents and pure red-cell aplasia: you can't fool Mother Nature. *Kidney International*.

[42] Halim L. A., Brinks V., Jiskoot W. (2014). How bio-questionable are the different recombinant human erythropoietin copy products in Thailand?. *Pharmaceutical Research*.

[43] Fotiou F., Aravind S., Wang P.-P., Nerapusee O. (2009). Impact of illegal trade on the quality of epoetin alfa in Thailand. *Clinical Therapeutics*.

[44] Bachmann M. F., Jennings G. T. (2010). Vaccine delivery: a matter of size, geometry, kinetics and molecular patterns. *Nature Reviews Immunology*.

[45] Wang M., Kaufman R. J. (2014). The impact of the endoplasmic reticulum protein-folding environment on cancer development. *Nature Reviews Cancer*.

[46] De Genst E., Messer A., Dobson C. M. (2014). Antibodies and protein misfolding: from structural research tools to therapeutic strategies. *Biochimica et Biophysica Acta*.

[47] Roberts C. J. (2014). Protein aggregation and its impact on product quality. *Current Opinion in Biotechnology*.

[48] Barnett G. V., Qi W., Amin S., Neil Lewis E., Roberts C. J. (2015). Aggregate structure, morphology and the effect of aggregation mechanisms on viscosity at elevated protein concentrations. *Biophysical Chemistry*.

[49] http://www.fda.gov/downloads/drugs/guidancecomplianceregulatoryinformation/guidances/ucm338856.pdf.

[50] Rosenberg A. S. (2006). Effects of protein aggregates: an immunologic perspective. *The AAPS Journal*.

[51] Bruhns P., Iannascoli B., England P. (2009). Specificity and affinity of human Fc*γ* receptors and their polymorphic variants for human IgG subclasses. *Blood*.

[52] Bajardi-Taccioli A., Blum A., Xu C., Sosic Z., Bergelson S., Feschenko M. (2015). Effect of protein aggregates on characterization of FcRn binding of Fc-fusion therapeutics. *Molecular Immunology B*.

[53] Perevozchikova T., Nanda H., Nesta D. P., Roberts C. J. (2015). Protein adsorption, desorption, and aggregation mediated by solid-liquid interfaces. *Journal of Pharmaceutical Sciences*.

[54] Phay M., Welzel A. T., Williams A. D. (2015). IgG Conformer's binding to amyloidogenic aggregates. *PLOS ONE*.

[55] Jelkmann W. (2012). Biosimilar recombinant human erythropoietins (“epoetins”) and future erythropoiesis-stimulating treatments. *Expert Opinion on Biological Therapy*.

[56] http://gabi-journal.net/wp-content/uploads/LatAM-SBP-2015-WS-V15B10-TP-FINAL1.pdf.

[57] Vincent F. B., Morand E. F., Murphy K., Mackay F., Mariette X., Marcelli C. (2013). Antidrug antibodies (ADAb) to tumour necrosis factor (TNF)-specific neutralising agents in chronic inflammatory diseases: a real issue, a clinical perspective. *Annals of the Rheumatic Diseases*.

[58] Jullien D., Prinz J. C., Nestle F. O. (2015). Immunogenicity of biotherapy used in psoriasis: the science behind the scenes. *Journal of Investigative Dermatology*.

[59] Armuzzi A., Lionetti P., Blandizzi C. (2014). anti-TNF agents as therapeutic choice in immune-mediated inflammatory diseases: focus on adalimumab. *International journal of immunopathology and pharmacology*.

[60] Smolen J. S., Landewé R., Breedveld F. C. (2014). EULAR recommendations for the management of rheumatoid arthritis with synthetic and biological disease-modifying antirheumatic drugs: 2013 update. *Annals of Rheumatic Diseases*.

[61] Wadhwa M., Knezevic I., Kang H.-N., Thorpe R. (2015). Immunogenicity assessment of biotherapeutic products: an overview of assays and their utility. *Biologicals*.

[62] Jefferis R., Lefranc M.-P. (2009). Human immunoglobulin allotypes: possible implications for immunogenicity. *mAbs*.

[63] Lefranc M.-P., Lefranc G. (2012). Human Gm, Km, and Am allotypes and their molecular characterization: a remarkable demonstration of polymorphism. *Methods in Molecular Biology*.

[64] Vidarsson G., Dekkers G., Rispens T. (2014). IgG subclasses and allotypes: from structure to effector functions. *Frontiers in Immunology*.

[65] Hmiel L. K., Brorson K. A., Boyne M. T. (2015). Post-translational structural modifications of immunoglobulin G and their effect on biological activity. *Analytical and Bioanalytical Chemistry*.

[66] WHO (2013). *Guidelines on the Quality, Safety, and Efficacy of Biotherapeutic Products Prepared by Recombinant DNA Technology*.

[67] EMA (2007). *Guideline on Immunogenicity Assessment of Biotechnology-Derived Therapeutic Proteins*.

[68] http://www.ema.europa.eu/docs/en_GB/document_library/Scientific_guideline/2012/06/WC500128688.pdf.

[69] FDA (2009). *Assay Development for Immunogenicity Testing of Therapeutic Proteins*.

[70] Mok C. C., Tsai W. C., Chen Y. (2015). Immunogenicity of anti-TNF biologic agents in the treatment of rheumatoid arthritis. *Expert Opinion in Biological Therapy*.

[71] Schaeverbeke T., Truchetet M., Kostine M., Barnetche T., Bannwarth B., Richez C. (2016). Immunogenicity of biologic agents in rheumatoid arthritis patients: lessons for clinical practice. *Rheumatology*.

[72] Edelman G. M., Cunningham B. A., Gall W. E., Gottlieb P. D., Rutishauser U., Waxdal M. J. (2004). The covalent structure of an entire gamma G immunoglobulin molecule. 1969. *Journal of Immunology*.

[73] Dickler H. B. (1974). Studies of the human lymphocyte receptor for heat aggregated or antigen complexed immunoglobulin. *Journal of Experimental Medicine*.

[74] Hunneyball I. M., Stanworth D. R. (1979). Studies on immune tolerance to heat-aggregated human IgG in rabbits: its relevance to the production of rheumatoid factors. *Immunology*.

[75] Gagnon P., Nian R., Leong D., Hoi A. (2015). Transient conformational modification of immunoglobulin G during purification by protein A affinity chromatography. *Journal of Chromatography A*.

[76] Telikepalli S. N., Kumru O. S., Kalonia C. (2014). Structural characterization of IgG1 mAb aggregates and particles generated under various stress conditions. *Journal of Pharmaceutical Sciences*.

[77] Filipe V., Poole R., Oladunjoye O., Braeckmans K., Jiskoot W. (2012). Detection and characterization of subvisible aggregates of monoclonal igg in serum. *Pharmaceutical Research*.

[78] Randolph T. W., Schiltz E., Sederstrom D. (2015). Do not drop: mechanical shock in vials causes cavitation, protein aggregation, and particle formation. *Journal of Pharmaceutical Sciences*.

[79] Freitag A. J., Shomali M., Michalakis S. (2015). Investigation of the immunogenicity of different types of aggregates of a murine monoclonal antibody in mice. *Pharmaceutical Research*.

[80] Chennamsetty N., Helk B., Voynov V., Kayser V., Trout B. L. (2009). Aggregation-prone motifs in human immunoglobulin G. *Journal of Molecular Biology*.

[81] Wang X., Das T. K., Singh S. K., Kumar S. (2009). Potential aggregation prone regions in biotherapeutics: a survey of commercial monoclonal antibodies. *mAbs*.

[82] Skamris T., Tian X., Thorolfsson M. (2016). Monoclonal antibodies follow distinct aggregation pathways during production-relevant acidic incubation and neutralization. *Pharmaceutical Research*.

[83] Huh J. H., White A. J., Brych S. R., Franey H., Matsumura M. (2013). The identification of free cysteine residues within antibodies and a potential role for free cysteine residues in covalent aggregation because of agitation stress. *Journal of Pharmaceutical Sciences*.

[84] Rouet R., Lowe D., Christ D. (2014). Stability engineering of the human antibody repertoire. *FEBS Letters*.

[85] Ewert S., Huber T., Honegger A., Plückthun A. (2003). Biophysical properties of human antibody variable domains. *Journal of Molecular Biology*.

[86] US Food and Drug Administration *Herceptin®, Avastin® and Remicade® Prescribing Information*.

[87] Arvinte T., Palais C., Green-Trexler E. (2013). Aggregation of biopharmaceuticals in human plasma and human serum: implications for drug research and development. *mAbs*.

[88] Luo S., Zhang B. (2015). Dextrose-mediated aggregation of therapeutic monoclonal antibodies in human plasma: implication of isoelectric precipitation of complement proteins. *mAbs*.

[89] Moore J. M. R., Patapoff T. W., Cromwell M. E. M. (1999). Kinetics and thermodynamics of dimer formation and dissociation for a recombinant humanized monoclonal antibody to vascular endothelial growth factor. *Biochemistry*.

[90] van Kasteren S. I., Overkleeft H., Ovaa H., Neefjes J. (2014). Chemical biology of antigen presentation by MHC molecules. *Current Opinion in Immunology*.

[91] Zhang W., Voice J., Lachman P. J. (1995). A systematic study of neutrophil degranulation and respiratory burst in vitro by defined immune complexes. *Clinical and Experimental Immunology*.

[92] Voice J. K., Lachmann P. J. (1997). Neutrophil Fc*γ* and complement receptors involved in binding soluble IgG immune complexes and in specific granule release induced by soluble IgG immune complexes. *European Journal of Immunology*.

[93] Steensgaard J., Jacobsen C., Lowe J., Ling N. R., Jefferis R. (1982). Theoretical and ultracentrifugal analysis of immune complex formation between monoclonal antibodies and human IgG. *Immunology*.

[94] Alduaij W., Ivanov A., Honeychurch J. (2011). Novel type II anti-CD20 monoclonal antibody (GA101) evokes homotypic adhesion and actin-dependent, lysosome-mediated cell death in B-cell malignancies. *Blood*.

[95] Niederfellner G., Lammens A., Mundigl O. (2011). Epitope characterization and crystal structure of GA101 provide insights into the molecular basis for type I/II distinction of CD20 antibodies. *Blood*.

[96] Kim M.-S., Lee S.-H., Song M.-Y., Yoo T. H., Lee B.-K., Kim Y.-S. (2007). Comparative analyses of complex formation and binding sites between human tumor necrosis factor-alpha and its three antagonists elucidate their different neutralizing mechanisms. *Journal of Molecular Biology*.

[97] Padlan E. A. (1996). X-ray crystallography of antibodies. *Advances in Protein Chemistry*.

[98] Guo D., Gao A., Michels D. A. (2010). Mechanisms of unintended amino acid sequence changes in recombinant monoclonal antibodies expressed in Chinese Hamster Ovary (CHO) cells. *Biotechnology and Bioengineering*.

[99] Nakajima C., Kuyama H., Nakazawa T., Nishimura O., Tsunasawa S. (2011). A method for N-terminal de novo sequencing of N*α*-blocked proteins by mass spectrometry. *Analyst*.

[100] Kumar A., Bachhawat A. K. (2012). Pyroglutamic acid: throwing light on a lightly studied metabolite. *Current Science*.

[101] Liu Y. D., Goetze A. M., Bass R. B., Flynn G. C. (2011). N-terminal glutamate to pyroglutamate conversion in vivo for human IgG2 antibodies. *The Journal of Biological Chemistry*.

[102] Yin S., Pastuskovas C. V., Khawli L. A., Stults J. T. (2013). Characterization of therapeutic monoclonal antibodies reveals differences between in vitro and in vivo time-course studies. *Pharmaceutical Research*.

[103] Perez-Garmendia R., Gevorkian G. (2013). Pyroglutamate-modified amyloid beta peptides: emerging targets for Alzheimer's disease immunotherapy. *Current Neuropharmacology*.

[104] Liu H., Ponniah G., Zhang H.-M. (2014). In vitro and in vivo modifications of recombinant and human IgG antibodies. *mAbs*.

[105] Merlini G., Comenzo R. L., Seldin D. C., Wechalekar A., Gertz M. A. (2014). Immunoglobulin light chain amyloidosis. *Expert Review of Hematology*.

[106] Li J., Menzel C., Meier D., Zhang C., Dübel S., Jostock T. (2007). A comparative study of different vector designs for the mammalian expression of recombinant IgG antibodies. *Journal of Immunological Methods*.

[107] Tang L., Sundaram S., Zhang J. (2013). Conformational characterization of the charge variants of a human IgG1 monoclonal antibody using H/D exchange mass spectrometry. *mAbs*.

[108] http://www.ebe-biopharma.eu/uploads/Modules/Documents/ebe-concept-paper-%E2%80%93-considerations-in-setting-specifications.pdf.

[109] Tsubaki M., Terashima I., Kamata K., Koga A. (2013). C-terminal modification of monoclonal antibody drugs: amidated species as a general product-related substance. *International Journal of Biological Macromolecules*.

[110] Škulj M., Pezdirec D., Gaser D., Kreft M., Zorec R. (2014). Reduction in C-terminal amidated species of recombinant monoclonal antibodies by genetic modification of CHO cells. *BMC biotechnology*.

[111] van den Bremer E. T. J., Beurskens F. J., Voorhorst M. (2015). Human IgG is produced in a pro-form that requires clipping of C-terminal lysines for maximal complement activation. *mAbs*.

[112] Liu H., May K. (2012). Disulfide bond structures of IgG molecules: structural variations, chemical modifications and possible impacts to stability and biological function. *mAbs*.

[113] Aricescu A. R., Owens R. J. (2013). Expression of recombinant glycoproteins in mammalian cells: towards an integrative approach to structural biology. *Current Opinion in Structural Biology*.

[114] Wypych J., Li M., Guo A. (2008). Human IgG2 antibodies display disulfide-mediated structural isoforms. *The Journal of Biological Chemistry*.

[115] Dillon T. M., Ricci M. S., Vezina C. (2008). Structural and functional characterization of disulfide isoforms of the human IgG2 subclass. *Journal of Biological Chemistry*.

[116] Koterba K. L., Borgschulte T., Laird M. W. (2012). Thioredoxin 1 is responsible for antibody disulfide reduction in CHO cell culture. *Journal of Biotechnology*.

[117] Hutterer K. M., Hong R. W., Lull J. (2013). Monoclonal antibody disulfide reduction during manufacturing: untangling process effects from product effects. *mAbs*.

[118] Kao Y. H., Laird M. W., Schmidt M. T., Wong R. L., Hewitt D. P. Prevention of disulfide bond reduction during recombinant production of polypeptides.

[119] Correia I. R. (2010). Stability of IgG isotypes in serum. *mAbs*.

[120] Rispens T., Meesters J., den Bleker T. H. (2013). Fc-Fc interactions of human IgG4 require dissociation of heavy chains and are formed predominantly by the intra-chain hinge isomer. *Molecular Immunology*.

[121] Davies A. M., Rispens T., den Bleker T. H. (2013). Crystal structure of the human IgG4 C_H_3 dimer reveals the role of Arg409 in the mechanism of Fab-arm exchange. *Molecular Immunology*.

[122] Rispens T., Davies A. M., Ooijevaar-de Heer P. (2014). Dynamics of inter-heavy chain interactions in human immunoglobulin G (IgG) subclasses studied by kinetic Fab arm exchange. *The Journal of Biological Chemistry*.

[123] Chumsae C., Gaza-Bulseco G., Sun J., Liu H. (2007). Comparison of methionine oxidation in thermal stability and chemically stressed samples of a fully human monoclonal antibody. *Journal of Chromatography B: Analytical Technologies in the Biomedical and Life Sciences*.

[124] Bertolotti-Ciarlet A., Wang W., Lownes R. (2009). Impact of methionine oxidation on the binding of human IgG1 to FcRn and Fc*γ* receptors. *Molecular Immunology*.

[125] Wang W., Vlasak J., Li Y. (2011). Impact of methionine oxidation in human IgG1 Fc on serum half-life of monoclonal antibodies. *Molecular Immunology*.

[126] Pan H., Chen K., Chu L., Kinderman F., Apostol I., Huang G. (2009). Methionine oxidation in human IgG2 Fc decreases binding affinities to protein A and FcRn. *Protein Science*.

[127] Gaza-Bulseco G., Faldu S., Hurkmans K., Chumsae C., Liu H. (2008). Effect of methionine oxidation of a recombinant monoclonal antibody on the binding affinity to protein A and protein G. *Journal of Chromatography B: Analytical Technologies in the Biomedical and Life Sciences*.

[128] Xie H., Chakraborty A., Ahn J. (2010). Rapid comparison of a candidate biosimilar to an innovator monoclonal antibody with advanced liquid chromatography and mass spectrometry technologies. *mAbs*.

[129] Chelius D., Render D. S., Bondarenko P. V. (2005). Identification and characterization of deamidation sites in the conserved regions of human immunoglobulin gamma antibodies. *Analytical Chemistry*.

[130] Liu Y. D., van Enk J. Z., Flynn G. C. (2009). Human antibody Fc deamidation in vivo. *Biologicals*.

[131] Sinha S., Zhang L., Duan S. (2009). Effect of protein structure on deamidation rate in the Fc fragment of an IgG1 monoclonal antibody. *Protein Science*.

[132] Huang L., Lu J., Wroblewski V. J., Beals J. M., Riggin R. M. (2005). In vivo deamidation characterization of monoclonal antibody by LC/MS/MS. *Analytical Chemistry*.

[133] Diepold K., Bomans K., Wiedmann M. (2012). Simultaneous assessment of Asp isomerization and Asn deamidation in recombinant antibodies by LC-MS following incubation at elevated temperatures. *PLoS ONE*.

[134] Vlasak J., Bussat M. C., Wang S. (2009). Identification and characterization of asparagine deamidation in the light chain CDR1 of a humanized IgG1 antibody. *Analytical Biochemistry*.

[135] Hansson K., Stenflo J. (2005). Post-translational modifications in proteins involved in blood coagulation. *Journal of Thrombosis and Haemostasis*.

[136] Fischer K., Iorio A., Lassila R. (2015). Inhibitor development in non-severe haemophilia across Europe. *Thrombosis and Haemostasis*.

[137] Powell J. S. (2014). Lasting power of new clotting proteins. *Hematology*.

[138] Grinnell B. W., Yan S. B., Macias W. L., McGrath B., Walsh G. (2006). Activated protein C. *Directory of Therapeutic Enzymes*.

[139] Liu J., Jonebring A., Hagström J., Nyström A.-C., Lövgren A. (2014). Improved expression of recombinant human factor IX by co-expression of GGCX, VKOR and furin. *Protein Journal*.

[140] Yang Y.-S., Wang C.-C., Chen B.-H., Hou Y.-H., Hung K.-S., Mao Y.-C. (2015). Tyrosine sulfation as a protein post-translational modification. *Molecules*.

[141] Darula Z., Chalkley R. J., Baker P., Burlingame A. L., Medzihradszky K. F. (2010). Mass spectrometric analysis, automated identification and complete annotation of O-linked glycopeptides. *European Journal of Mass Spectrometry*.

[142] Costagliola S., Panneels V., Bonomi M. (2002). Tyrosine sulfation is required for agonist recognition by glycoprotein hormone receptors. *The EMBO Journal*.

[143] McGrath B., McGrath B., Walsh G. (2006). Factor IX (protease zymogen). *Directory of Therapeutic Enzymes*.

[144] Ludeman J. P., Stone M. J. (2014). The structural role of receptor tyrosine sulfation in chemokine recognition. *British Journal of Pharmacology*.

[145] Choe H., Li W., Wright P. L. (2003). Tyrosine sulfation of human antibodies contributes to recognition of the CCR5 binding region of HIV-1 gp120. *Cell*.

[146] Llop E., Gallego R. G., Belalcazar V. (2007). Evaluation of protein N-glycosylation in 2-DE: erythropoietin as a study case. *Proteomics*.

[147] Yin B., Gao Y., Chung C.-Y. (2015). Glycoengineering of Chinese hamster ovary cells for enhanced erythropoietin N-glycan branching and sialylation. *Biotechnology and Bioengineering*.

[148] Jefferis R., Davis D. L., Schiel J., Borisov O. (2014). Monoclonal antibodies: mechanisms of action. *Current State of the Art & Emerging Technologies for the Characterisation of Monoclonal Antibodies, Volume 1. Monoclonal Antibody Therapeutics: Structure, Function, and Regulatory Space*.

[149] Brady L. J., Velayudhan J., Visone D. B. (2015). The criticality of high-resolution N-linked carbohydrate assays and detailed characterization of antibody effector function in the context of biosimilar development. *mAbs*.

[150] Reusch D., Tejada M. L. (2015). Fc glycans of therapeutic antibodies as critical quality attributes. *Glycobiology*.

[151] Liu L. (2015). Antibody glycosylation and its impact on the pharmacokinetics and pharmacodynamics of monoclonal antibodies and Fc-fusion proteins. *Journal of Pharmaceutical Sciences*.

[152] Mimura Y., Ashton P. R., Takahashi N., Harvey D. J., Jefferis R. (2007). Contrasting glycosylation profiles between Fab and Fc of a human IgG protein studied by electrospray ionization mass spectrometry. *Journal of Immunological Methods*.

[153] Masuda K., Kubota T., Kaneko E. (2007). Enhanced binding affinity for Fc*γ*RIIIa of fucose-negative antibody is sufficient to induce maximal antibody-dependent cellular cytotoxicity. *Molecular Immunology*.

[154] Raju T. S., Lang S. E. (2014). Diversity in structure and functions of antibody sialylation in the Fc. *Current Opinion in Biotechnology*.

[155] Thomann M., Schlothauer T., Dashivets T. (2015). In vitro glycoengineering of IgG1 and its effect on Fc receptor binding and ADCC activity. *PLoS ONE*.

[156] Heffner K. M., Hizal D. B., Kumar A. (2014). Exploiting the proteomics revolution in biotechnology: from disease and antibody targets to optimizing bioprocess development. *Current Opinion in Biotechnology C*.

[157] van Berkel P. H. C., Gerritsen J., Perdok G. (2009). N-linked glycosylation is an important parameter for optimal selection of cell lines producing biopharmaceutical human IgG. *Biotechnology Progress*.

[158] Hossler P. (2012). Protein glycosylation control in mammalian cell culture: past precedents and contemporary prospects. *Advances in Biochemical Engineering/Biotechnology*.

[159] McCracken N. A., Kowle R., Ouyang A. (2014). Control of galactosylated glycoforms distribution in cell culture system. *Biotechnology Progress*.

[160] Daguet A., Watier H. (2011). 2nd Charles Richet et Jules Héricourt workshop: therapeutic antibodies and anaphylaxis; May 31-June 1, 2011; Tours, France. *mAbs*.

[161] Platts-Mills T. A. E., Schuyler A. J., Tripathi A., Commins S. P. (2015). Anaphylaxis to the carbohydrate side chain alpha-gal. *Immunology and Allergy Clinics of North America*.

[162] Galili U. (2013). Discovery of the natural anti-Gal antibody and its past and future relevance to medicine. *Xenotransplantation*.

[163] Bosques C. J., Collins B. E., Meador J. W. (2010). Chinese hamster ovary cells can produce galactose-*α*-1,3-galactose antigens on proteins. *Nature Biotechnology*.

[164] Mimura Y., Church S., Ghirlando R. (2001). The influence of glycosylation on the thermal stability and effector function expression of human IgG1-Fc: properties of a series of truncated glycoforms. *Molecular Immunology*.

[165] Mimura Y., Sondermann P., Ghirlando R. (2001). Role of oligosaccharide residues of IgG1-Fc in Fc*γ*RIIb Binding. *The Journal of Biological Chemistry*.

[166] Krapp S., Mimura Y., Jefferis R., Huber R., Sondermann P. (2003). Structural analysis of human IgG-Fc glycoforms reveals a correlation between glycosylation and structural integrity. *Journal of Molecular Biology*.

[167] Ha C., Mathur J., Kornbluth A. (2015). Anti-TNF levels and anti-drug antibodies, immunosuppressants and clinical outcomes in inflammatory bowel disease. *Expert Review of Gastroenterology and Hepatology*.

[168] Choe J.-Y., Prodanovic N., Niebrzydowski J. (2015). A randomised, double-blind, phase III study comparing SB2, an infliximab biosimilar, to the infliximab reference product Remicade in patients with moderate to severe rheumatoid arthritis despite methotrexate therapy. *Annals of the Rheumatic Diseases*.

[169] Mitchison N. A. (1964). Induction of immunological paralysis in two zones of dosage. *Proceedings of the Royal Society of London. Series B, Containing Papers*.

[170] Somerfield J., Hill-Cawthorne G. A., Lin A. (2010). A novel strategy to reduce the immunogenicity of biological therapies. *The Journal of Immunology*.

[171] Tito M. A., Miller J., Walker N. (2001). Probing molecular interactions in intact antibody: antigen complexes, an electrospray time-of-flight mass spectrometry approach. *Biophysical Journal*.

[172] Atmanene C., Wagner-Rousset E., Malissard M. (2009). Extending mass spectrometry contribution to therapeutic monoclonal antibody lead optimization: characterization of immune complexes using noncovalent ESI-MS. *Analytical Chemistry*.

[173] Atmanene C., Wagner-Rousset E., Corvaïa N., Van Dorsselaer A., Beck A., Sanglier-Cianférani S. (2013). Noncovalent mass spectrometry for the characterization of antibody/antigen complexes. *Methods in Molecular Biology*.

[174] Oda M., Uchiyama S., Noda M. (2009). Effects of antibody affinity and antigen valence on molecular forms of immune complexes. *Molecular Immunology*.

[175] Thompson N. J., Rosati S., Heck A. J. R. (2014). Performing native mass spectrometry analysis on therapeutic antibodies. *Methods*.

[176] Thompson N. J., Rosati S., Rose R. J., Heck A. J. R. (2013). The impact of mass spectrometry on the study of intact antibodies: from post-translational modifications to structural analysis. *Chemical Communications*.

